# Investigating Mirror System (MS) Activity in Adults with ASD When Inferring Others’ Intentions Using Both TMS and EEG

**DOI:** 10.1007/s10803-018-3492-2

**Published:** 2018-02-16

**Authors:** Eleanor J. Cole, Nick E. Barraclough, Peter G. Enticott

**Affiliations:** 1 The Department of Psychology, The University of York, Heslington, York, North Yorkshire YO10 5DD UK; 2 Cognitive Neuroscience Unit, Faculty of Health, Deakin University Burwood Campus, 221 Burwood Highway, Melbourne, VIC 3125 Australia

**Keywords:** Autism spectrum disorder (ASD), Mirror system (MS), Mentalizing, Transcranial magnetic stimulation (TMS), Electroencephalography (EEG), Intentions

## Abstract

ASD is associated with mentalizing deficits that may correspond with atypical mirror system (MS) activation. We investigated MS activity in adults with and without ASD when inferring others’ intentions using TMS-induced motor evoked potentials (MEPs) and mu suppression measured by EEG. Autistic traits were measured for all participants. Our EEG data show, high levels of autistic traits predicted reduced right mu (8–10 Hz) suppression when mentalizing. Higher left mu (8–10 Hz) suppression was associated with superior mentalizing performances. Eye-tracking and TMS data showed no differences associated with autistic traits. Our data suggest ASD is associated with reduced right MS activity when mentalizing, TMS-induced MEPs and mu suppression measure different aspects of MS functioning and the MS is directly involved in inferring intentions.

**Electronic supplementary material:**

The online version of this article (10.1007/s10803-018-3492-2) contains supplementary material, which is available to authorized users.

## Introduction

Experimental evidence and anecdotal reports suggest that individuals with autism spectrum disorder (ASD) diagnoses display difficulties inferring the thoughts, feeling and beliefs of others, collectively known as ‘mentalizing’ (Baron-Cohen et al. 1997; Castelli et al. 2002; Jolliffe and Baron-Cohen 1999; Kana et al. 2014; Senju et al. 2009). ASD is a term used by the most recent edition of the Diagnostic and Statistical Manual of Mental Disorders (DSM-5) to describe a number of neurodevelopmental disorders characterised by difficulties in social communication as well as restricted and repetitive behaviours (American Psychiatric Association 2013). Due to the spectral nature of ASD, individuals with and without ASD diagnoses display varying degrees of autistic traits. Individuals without an ASD diagnosis but who display relatively high levels of autistic traits have also been shown to exhibit mentalizing deficits (Gökçen et al. 2014, 2016).

The ‘broken mirror’ theory of ASD suggests that dysfunction in brain areas known collectively as the mirror system (MS) underlie some of the social communication difficulties experienced by individuals with ASD (Iacoboni and Dapretto 2006; Oberman and Ramachandran 2007). The main components of the human MS are considered to be the inferior frontal gyrus (IFG) and the inferior parietal lobe (IPL; Rizzolatti and Craighero 2004; Rizzolatti and Sinigaglia 2010). Areas of the MS are active during the performance of an action as well as the observation of similar actions (di Pellegrino et al. 1992; Rizzolatti et al. 1996). It is thought that by displaying similar activation patterns during the observation of actions as when performing actions, the MS simulates observed actions in the observer’s own motor system to facilitate action understanding (Rizzolatti and Craighero 2004). This is known as the motor resonance theory (Agnew et al. 2007; Landmann et al. 2011; Leslie et al. 2004; Rizzolatti et al. 2002). According to the broken mirror theory, atypical MS activation in individuals with ASD results in reduced understanding of the actions of others, which in turn underlies some of the social communication difficulties these individuals experience (Iacoboni and Dapretto 2006; Oberman and Ramachandran 2007).

Although the broken mirror hypothesis is an attractive theory, the literature supporting the possibility of atypical MS activation in individuals with ASD is limited, particularly in adults. A number of behavioural studies, fMRI studies and studies using electromyographic recordings have shown children with ASD display both behavioural impairments and atypical MS activity during tasks typically associated with MS functioning such as imitation (Dapretto et al. 2006; Hobson and Hobson 2008; Rogers et al. 2003; Williams et al. 2004), action planning (Cattaneo et al. 2007; Dowd et al. 2012; Fabbri-Destro et al. 2009) and gestural performance (Dewey et al. 2007). In contrast, adults with ASD generally display typical behavioural performances on tasks traditionally associated with MS functioning (Bird et al. 2007; Avikainen et al. 2003) and the majority of neuroimaging studies (fMRI, EEG and TMS) have shown that adults with ASD display typical levels of MS activity (Avikainen et al. 1999; Dinstein et al. 2010; Enticott et al. 2013b; Marsh and Hamilton 2011). Only a limited number of studies have provided evidence to suggest MS activation is atypical during these tasks in adults with ASD (Bernier et al. 2007; Enticott et al. 2012; Honaga et al. 2010; Martineau et al. 2010) and adults with high levels of autistic traits (Cooper et al. 2013; Lepage et al. 2010; Puzzo et al. 2009). Therefore, evidence to support general dysfunction of the MS in ASD, particularly in adults, is limited (Hamilton 2013).

Despite the limited evidence suggesting atypical MS activity in adults with ASD during tasks traditionally associated with MS functioning (e.g. imitation and action planning), fMRI studies have found reduced MS activation (IFG and IPL) in adults with ASD during mentalizing tasks compared to control participants (Baron-Cohen et al. 1999; Holt et al. 2014; Kana et al. 2014). A wide body of neuroimaging literature has provided evidence for MS involvement in mentalizing in typically developing adults: higher MS activity has been shown during mentalizing tasks than non-mentalizing tasks (fMRI; Adams et al. 2010; Centelles et al. 2011; de Lange et al. 2008; Schurz et al. 2014; PET; Brunet et al. 2000). Additionally, both fMRI and TMS studies have found higher MS activation during the observation of actions with social context compared to non-social actions even in the absence of mentalizing tasks (Bucchioni et al. 2013; Ciaramidaro et al. 2014; Enticott et al. 2013b; Iacoboni et al. 2004). Lesions to IFG, both in brain damaged patients (Besharati et al. 2016; Dal Monte et al. 2014) and when temporary functional lesions are induced via direct current stimulation in patients undergoing surgery to treat epilepsy (Herbet et al. 2014), have been shown to impair mentalizing performances. Collectively, these data show that MS has a role in mentalizing and that MS functioning is atypical in adults with ASD when the mentalizing system is engaged. Therefore, it is possible that reduced MS activity during mentalizing tasks may contribute to the mentalizing difficulties these adults experience.

Despite numerous studies providing evidence for a role of the MS in mentalizing, some fMRI studies have not found higher levels of MS activity during mentalizing tasks compared to non-mentalizing tasks in typically developing participants (Castelli et al. 2000, 2002; Gallagher et al. 2000; Spunt et al. 2011; White et al. 2014). Differences in the stimuli used are likely to have contributed to inconsistencies in the existing literature. fMRI studies have shown that different brain areas are active during mentalizing tasks depending on the stimuli used (Gobbini et al. 2007; Schurz et al. 2014), higher MS activation is elicited when dynamic stimuli are used rather than static stimuli and when stimuli depict bodies rather than faces (Schlochtermeier et al. 2015). The majority of mentalizing tasks that have not elicited MS activation have used simplistic cartoons, still images or passages of text as stimuli (Castelli et al. 2000, 2002; Gallagher et al. 2000; White et al. 2014). If MS functioning is atypical in adults with ASD during mentalizing tasks then these individuals may display more prominent differences in brain activation and greater behavioural impairments on mentalizing tasks that typically elicit greater levels of MS activity.

Transcranial magnetic stimulation (TMS) and electroencephalography (EEG) are two techniques that have often been used to non-invasively obtain indirect indices of MS activity, but it is unknown precisely how these two indices of MS activity relate to each other. TMS involves administering brief magnetic pulses through a magnetic coil placed on the scalp in order to induce transient changes in activity in the underlying region of the cortex (Hallett 2000). When single TMS pulses are applied to the primary motor cortex (M1), the resulting increases in corticospinal activity can be measured by recording increased activity in contralateral hand muscles via electromyography (EMG; Aziz-Zadeh et al. 2002; Fadiga et al. 2005). These increases in muscle activity induced by TMS (known as motor evoked potentials; MEPs) are larger when individuals view hand actions compared to when TMS is applied at rest and therefore these increases in MEP sizes during action observation are regarded as an index of MS activity (Fadiga et al. 2005; Patuzzo et al. 2003; Strafella and Paus 2000). In contrast, *mu* rhythm; large amplitude oscillations in the alpha frequency band (8–12 Hz) over sensorimotor cortex detected by EEG, is suppressed during action observation as well as the performance of actions and thereby provides another index of MS activity (Fox et al. 2016; Frenkel-Toledo et al. 2014; Oberman et al. 2007). Two previous studies that have combined EEG and single-pulse TMS to measure MS activity in typically developing populations have shown that although measurements from both these techniques are sensitive to motor resonance mechanisms, they are not correlated with each other (Andrews et al. 2015; Lepage et al. 2008). Therefore, it is possible that these measurements reflect different aspects of MS functioning. It is important to note that these indices of MS activity also differ in their spatial and temporal properties; EEG measures the sum of post-synaptic neuronal activity over a large area, and an index of mu suppression is typically taken over a relatively long time period (i.e., > 1 s). By contrast, TMS measures brief induced increases in corticospinal activity from peripheral muscles (Andrews et al. 2015; Pineda 2005; Rossini et al. 1994). Using both of these non-invasive measures of MS activity simultaneously allows a more complete picture of MS functioning to be collected.

This study aimed to investigate whether adults with diagnoses of ASD display atypical MS activity when mentalizing and whether levels of MS activation correspond to mentalizing performance. In this study, participants watched hand action videos, and after each video they had to either make decisions about the intention of the actor (mentalizing task) or the success of the action (non-mentalizing task). The video stimuli used showed different actors performing naturalistic hand actions to ensure the stimuli were sufficiently complex and optimally activated the MS. TMS-induced MEPs and *mu* suppression were both used as indices of MS activity. A preliminary TMS study carried out on typically developing individuals, using the same stimuli, identified higher MS activation during a mentalizing task compared to a non-mentalizing task once the actors’ intentions had been revealed (Cole and Barraclough 2017). Therefore, in our study, single-pulse TMS was applied to the primary motor cortex (M1) at the end of each hand action when the outcome of the action or the intention of the actor had been revealed. Simultaneous EEG recordings were made throughout the experiment. Participants without a diagnosis were grouped based on low or high levels of autistic traits. It was predicted that larger TMS-induced MEP sizes and greater levels of *mu* suppression would be found during the mentalizing task compared to the non-mentalizing task, indicating higher levels of MS activity. It was also predicted that high levels of autistic traits would be associated with reduced task-related changes in MS activity and that lower levels of MS activity would be related to poorer mentalizing performances.

## Methods

### Participants

Forty-three adults were recruited for this study, of which 13 had a formal diagnosis of either Asperger’s disorder (11) or ASD. All of the participants with a diagnosis met the DSM-5 criteria for ASD and none of the participants had any existing learning difficulties or experienced delayed language development. Participants without an ASD diagnosis were recruited based on the level of autistic traits they displayed as measured by the Autistic Spectrum Quotient (AQ; Baron-Cohen et al. 2001). The average AQ score in the general population is 16.94 (Ruzich et al. 2015). Individuals were excluded from the study if they had AQ scores between 16 and 19. Participants with scores between 0 and 15 were assigned to the ‘low AQ’ group and participants with AQ scores of 20 or higher were assigned to the ‘high AQ’ group. This resulted in three participant groups: low AQ (n = 15), high AQ (n = 15) and ASD (n = 13). Participants without an ASD diagnosis were grouped into low and high AQ groups due to findings of subtler versions of the behavioural and neural characteristics associated with ASD in individuals without a diagnosis but relatively high levels of autistic traits (Best et al. 2015; Di Martino et al. 2009; Lindell et al. 2009; Ridley et al. 2011; van Boxtel and Lu 2013). AQ scores were used to group these individuals initially and further psychological assessments were later used to quantify the level of autistic traits displayed in more detail (see Psychological Assessments). The participant groups did not significantly differ in age, verbal IQ, gender or years of formal education and all participants had verbal IQ scores within the normal range (> 70; see Table 1).

**Table 1:** Demographic information; group mean (SD) values

	Low AQ	High AQ	ASD	p	η_p_^2^
N	15	15	13		
Age	23.40 (6.82)	24.13(4.68)	28.30 (9.40)	0.16	0.09
Gender (m:f)	8:7	7:8	9:4	0.47 (X^2^)	/
Years of formal education	15.60 (1.64)	16.20 (1.66)	15.38 (1.45)	0.37	0.05
Verbal IQ^a^	109.67 (14.09)	113.00 (9.22)	111.62 (14.98)	0.78	0.01

p values were obtained from one-way MANOVA unless otherwise stated

^a^The verbal IQ scores were measured using the test of pre-morbid functioning

All participants were screened for symptoms of psychiatric disorders using the Mini-International Neuropsychiatric Interview (MINI) (Sheehan et al. 1998). Individuals were not eligible to take part if they were diagnosed with any psychiatric disorders or were identified by the MINI as displaying symptoms of any psychiatric disorders. An exception was made for mood disorders, anxiety and ADHD in the participants with ASD due to the high prevalence of these comorbidities (Matson et al. 2013; Matson and Williams 2014). In the ASD group, six participants were taking psychotropic medication to treat ADHD, depression or anxiety (see Table 2). None of the participants without an ASD diagnosis were taking psychotropic medication.

**Table 2:** Medication information for participants in the ASD group

Participant	Medication (daily dosage)
1	Dexamphetamine (20 mg), Zoloft (150 mg)
2	Dexamphetamine (20 mg)
3	Ritalin (30 mg)
4	Valium (5 mg when needed)
5	Zoloft (50 mg)
6	Zoloft (50 mg)

Participants were also screened for contraindications for TMS; history of seizures, serious head injuries, brain related conditions, severe headaches, implanted metal or medical devices, family history of epilepsy and current pregnancy (Rossi et al. 2009).

This research project was approved by the Human Research Ethics Committee at Deakin University and was performed in accordance with the ethical standards laid down in the 1990 Declaration of Helsinki. All participants provided signed informed consent.

### Psychological Assessments

All participants completed the Autism Spectrum Quotient (AQ), Autism Diagnostic Observation Schedule (ADOS-2), The Awareness of Social Inference Test (TASIT), Social Responsiveness Scale (SRS-2) and Empathy Quotient (EQ). The AQ and ADOS-2 are designed to measure the level of autistic traits displayed, the SRS-2 and TASIT measure social functioning and the EQ provides a measure of empathy. The three groups significantly differed from each other on all these measures (see Table 3). These psychological tests have been shown to display good psychometric properties (Allison et al. 2011; Hurst et al. 2007; McDonald et al. 2006; Oosterling et al. 2010). The AQ was administered in the form of an online questionnaire before the participants took part in the experiment. The other assessments were administered at the Cognitive Neuroscience Unit at Deakin University as a 2-h session prior to the TMS testing session. Psychological assessment sessions always took place on a separate date to the TMS session and both sessions were completed within a 2 week time frame.

**Table 3:** Participants’ psychological test scores; group mean (SD) values

	Low AQ	High AQ	ASD	p	η_p_^b^
AQ	8.80 (4.38)	24.07 (4.27)	32.00 (6.67)	< 0.001	0.79
EQ	53.07 (11.82)	42.60 (13.61)	27.23 (12.09)	< 0.001	0.43
TASIT^a^	58.73 (3.10)	54.07 (4.13)	52.38 (5.49)	0.001	0.30
ADOS-2	1.40 (0.99)	4.67 (1.40)	8.54 (3.28)	< 0.001	0.78
SRS-2^b^	27.87 (15.70)	58.60 (19.10)	101.46 (26.00)	< 0.001	0.69

^a^The TASIT scores were obtained from part 3 (social inference test)

^b^The reported SRS-2 scores are the unstandardized, raw scores, where scores above 60 indicate some social impairment and scores above 75 reflect severe social impairment

### Experimental Set-Up

Participants sat 600 mm away from an Eyelink 1000 plus eye-tracker (SR Research, Ontario) placed in front of a 24″ LED computer monitor. Single EEG electrodes were placed at locations FCz, F3 and F4 according to the international 10–20 system of electrode placement. Typically, EEG recordings are taken from central electrodes (C3, C4, Cz) when investigating MS activity. However, due to the placement of the TMS coil over the primary motor cortex (M1) EEG recordings were taken from frontal electrodes (F3, F4, FCz) to reduce TMS-induced artefacts, and to allow sufficient contact between the TMS coil and the scalp. It has previously been shown that *mu* suppression can be measured across the entire scalp when observing and imitating hand actions and ASD participants have been shown to display differences in *mu* power over frontal regions (Dumas et al. 2014). Reference electrodes were placed on the left and right mastoid bones and the ground electrode was placed on the forehead. Electrooculogram (EOG) electrodes were placed above and below the left eye to in order to identify EEG artefacts caused by blinking. EEG signals were recorded using Curry Neuroimaging Suite 7 (Compumetics Ltd, Australia). EEG signals were amplified using NeuroScan SynAmps RT (NeuroScan SynAmps, Compumedics Ltd.) and digitised at 1 kHz. All electrode impedances were below 5 KΩ. EEG analyses and bandpass filtering were conducted offline.

TMS was administered using a Magstim BiStim^2^ stimulator (Magstim Company Ltd., Carmarthenshire, Wales, UK). Firstly, the location of the primary motor cortex (M1) was identified in each participant by measuring the position on the scalp five centimetres lateral and 1 cm anterior to Cz (according to the international 10–20 system of electrode placement). TMS pulses were then applied to this position on the scalp using a standard figure of eight 70 mm coil held tangentially over the scalp at a 45° angle to the midline. An initial intensity of 40% stimulator output was used and then the intensity of TMS stimulation was increased in 5% increments until MEPs were produced. Stimulation was also applied around the estimated location of M1 in order to confirm that this was the optimal scalp location to produce MEPs in muscles of the right hand. MEPs were measured from the first dorsal interosseous (FDI) and abductor digiti minimi (ADM) muscles using Ag/AgCl surface electrodes. EMG signals were amplified using PowerLab 4/35 (with dual BioAmp; AD Instruments, Colorado Springs, CO). Once the optimal location for stimulation had been identified, the intensity of the TMS stimulation was adjusted in order to find the participants’ resting motor threshold (RMT). Participants’ RMT was defined as the minimum stimulation intensity needed to induce MEPs with an average peak-to-peak magnitude of 1 mV over 5 consecutive trials. Twenty MEPs induced by stimulation at RMT were used as a measure of baseline corticospinal excitability (CSE).

### Experimental Task

**Fig. 1:**
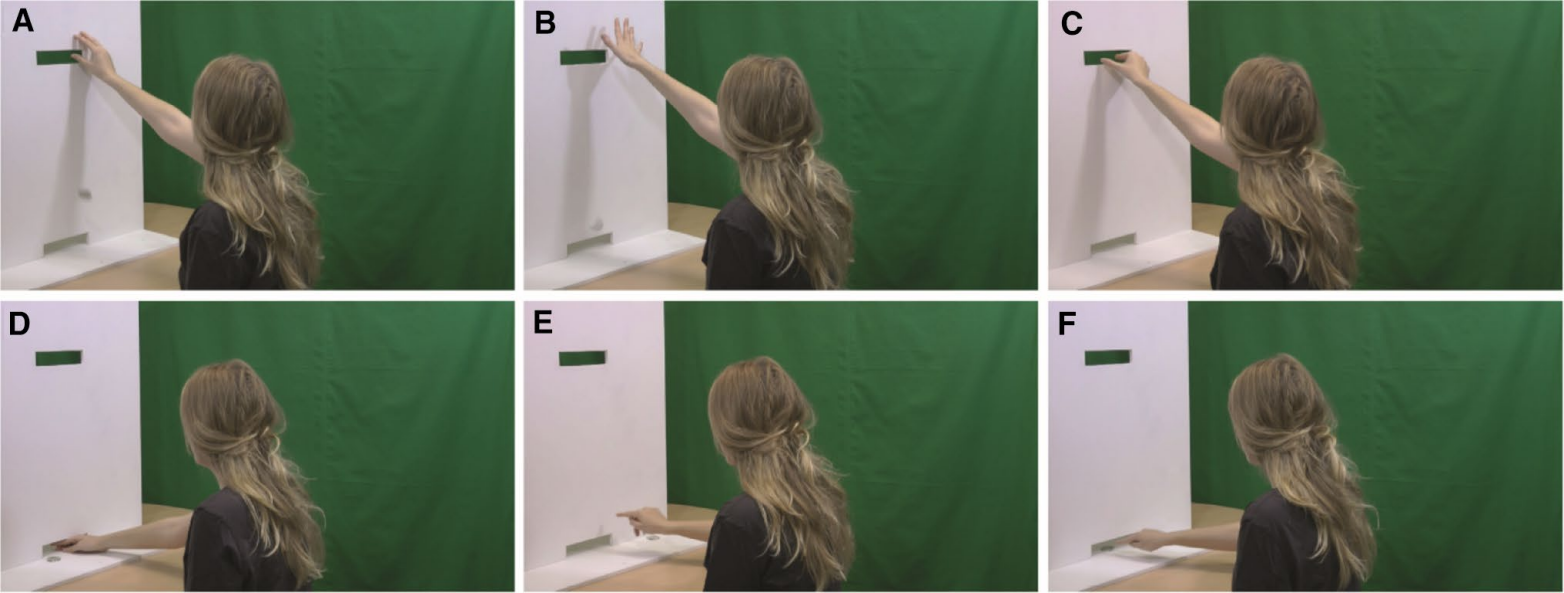
Screenshots depicting the final frame of the video stimuli for one actor. The videos depict an actor: **a** Accidently dropping a poker chip (clumsy grasp). **b** Deliberately dropping a poker chip (spiteful grasp). **c** Passing a poker chip through the higher slot in the board (successful grasp). **d** Accidentally not passing a poker chip through the bottom slot (clumsy push). **e** Deliberately not passing a poker chip through the bottom slot (spiteful push). **f** Passing a poker chip through the bottom slot (successful push)

The experiment comprised of two blocks; a mentalizing task and a non-mentalizing task. In both tasks, participants watched short videos (4 s) of actors passing or attempting to pass a poker chip through slots in a wooden board to another person on the other side of the board (who was out of view; see Fig. 1). After each video, participants were asked to make a decision about the action; either about the intention of the actor (mentalizing task) or the success of the action (non-mentalizing task). Participants indicated their response by pressing buttons on the computer keyboard with their left hand. In the mentalizing task, participants watched videos that either showed an actor accidentally dropping a poker chip and therefore failing to pass the poker chip to the other player (‘clumsy’ action) or an actor deliberately not passing the poker chip (‘spiteful’ action). After each video, participants indicated whether they thought the action was ‘clumsy’ or ‘spiteful’. In the non-mentalizing block, participants watched videos in which actors either successfully passed the poker chip to the other player (successful action) or accidentally dropped the poker chip (unsuccessful action). After each video, participants had to indicate whether the action was ‘successful’ or ‘unsuccessful’. The unsuccessful actions shown in the non-mentalizing block were the same as the ‘clumsy’ actions shown in the mentalizing block. Before each block, participants were told which decision they would be required to make after each video in the upcoming block (the instructions given to participants are available in the supplementary material). Participants also completed 16 practice trials (8 mentalizing trials) before the main experiment so that they knew what the tasks would entail. The actors shown in the practice trials were not shown in the main experiment to avoid any preconceptions about the actors influencing decisions made in the main experiment. The videos shown in this experiment are a subset of the videos used in a previous study (Cole et al. 2017). Grasping and pushing actions were shown; grasping actions involved actors grasping a poker chip and placing it through a slot at head height, pushing actions involved pushing the poker chip with the index finger through a slot that was level with the table in front of them (see Fig. 1). Twenty actors (10 male) were shown performing each action (clumsy grasp, clumsy push, spiteful grasp, spiteful push, successful grasp, successful push) resulting in 80 action videos in each of the two blocks (clumsy actions were seen in both blocks). Two types of actions (grasping and pushing) were used in order to make the stimuli more varied and these particular actions were chosen as they both utilise the FDI muscle.

A single TMS pulse at 1 mV RMT was delivered at the end of each video. A light sensor was used in order to time lock the TMS pulses to the timing of the videos. A black square was added to the top left corner of the videos and this black square was replaced with a white square for the last three frames in each video. The light sensor detected this change and sent a 5 V TTL pulse to the TMS stimulator via a BNC cable which triggered a single TMS pulse to be fired. The TMS machine subsequently sent a trigger to a PowerLab 4/35 (ADInstruments Pty Ltd) to trigger EMG recording. EEG was continuously recorded throughout both the mentalizing and non-mentalizing tasks but triggers were sent to the EEG machine at the start of each trial to record the type of action being shown. The order in which mentalizing and non-mentalizing blocks were completed was counterbalanced across all participants and within each participant group. Once participants had completed both the mentalizing and the non-mentalizing task, twenty single TMS pulses were administered at RMT in order to compare baseline corticospinal excitability before and after the experiment.

### Behavioural Analysis

First, the ADOS-2, AQ, EQ, SRS-2 and TASIT scores were calculated and a one-way MANOVA was used to identify group differences in these scores. Then, the numbers of correct responses on the mentalizing and non-mentalizing tasks were calculated for each participant. Data screening identified that the behavioural data were not normally distributed and therefore a log transformation was applied. The log transformed data still violated the assumption of normality so non-parametric analyses were conducted. Potential group differences in behavioural performances were explored using a Kruskal–Wallis test and a possible task-related difference in performances across all participants was examined using a Wilcoxon signed rank test.

### EMG Analysis

TMS was not performed on two participants in the high AQ group and four participants in the ASD group; two participants in the ASD group and one participant in the high AQ group found TMS too uncomfortable and the remaining three participants had motor thresholds deemed too high to continue (> 75% stimulator output). Trials in which muscle activity (± 0.1 mv) was identified within a 200 ms time window before the TMS pulse or trials in which FDI peak to peak MEP amplitudes were smaller than 0.2 mV were removed from the analysis (4.02% of all MEPs were excluded). Two participants in the high AQ group were removed from the analyses for having only 50% or fewer valid FDI MEPs for either task. This resulted in 35 participants (15 low AQ, 11 high AQ and 9 ASD) being included in the EMG analysis.

Preliminary analyses were carried out on the EMG data in order to clarify that RMTs were not significantly different between groups, that the experiment did not alter participants’ resting corticospinal activity and that the number of excluded MEPs did not significantly differ across tasks or participant groups. Group differences in RMTs were investigated using a one-way ANOVA. Changes in corticospinal activity as a result of the experiment were investigated by first calculating median MEP sizes (peak-to-peak amplitude [mV]) for both the 20 single TMS pulses given before the experiment and after the experiment for both muscles. Then, separate mixed-model ANOVAs were performed for each muscle investigating the influences of group (low AQ, high AQ, ASD) and time point (before or after the experiment) on MEP sizes. The data regarding the number of excluded MEPs violated the assumption of normality even after a log transformation was applied so non-parametric tests were used. An independent-samples Kruskal–Wallis test was used to investigate group differences and a related-samples Wilcoxon signed rank test was used to investigate differences in the number of MEPs excluded between tasks.

For the main TMS data analyses, median MEP values were calculated for both the FDI and the ADM muscles for each participant and each task (mentalizing/non-mentalizing). Median baseline MEP values were also calculated for both muscles for each participant by combining MEPs from both pre- and post-experiment baseline measures. The raw median MEP values for each task were then converted into motor resonance values by computing the relative MEP sizes in comparison to MEP sizes at baseline:

$${\text{MR}}=\left[ {\left( {{\text{median}}\;{\text{MEP}}\;{\text{during}}\;{\text{task}} - {\text{median}}\;{\text{MEP}}\;{\text{at}}\;{\text{baseline}}} \right)/{\text{median}}\;{\text{MEP}}\;{\text{at}}\;{\text{baseline}}} \right]*100$$

Data screening found that the motor resonance data for both FDI and ADM muscles violated the assumption of normality so a log transformation was used. This transformation cannot be performed on negative values so a constant of 100 was added to each motor resonance value prior to transformation to ensure that all values were positive. After the log transformation, the distribution of the FDI data did not significantly differ from a normal distribution but the ADM data still violated the assumption of normality. Therefore, parametric analyses were used for the log transformed FDI data, but non-parametric analyses were conducted on the log transformed ADM muscle data.

The FDI motor resonance data were analysed using a mixed-model ANOVA to investigate the influences of group (low AQ, high AQ, ASD) and task (mentalizing/non-mentalizing) on MEP sizes. Potential group differences in the ADM motor resonance data were investigated using a Kruskal–Wallis test and potential differences in motor resonances across experimental tasks were explored using a Wilcoxon signed rank test.

### EEG Analysis

Offline analyses of the EEG data were performed using Curry 7 Neuroimaging Suite software (Compumetics Ltd, Australia). Epochs of EEG data were created for videos shown in each task (mentalizing and non-mentalizing). Although, the action videos were 4000 ms long, the last 350 ms of each epoch was removed in order to eliminate the artefact created by the TMS pulse. Therefore, each video epoch was 3650 ms long and 80 epochs of each type were created for every participant. EEG data collected when participants were viewing a fixation cross were used as a baseline measure. There were 160 fixation cross epochs (80 for each task), each 1500 ms long. The first 500 ms of each fixation cross epoch were removed from the analysis because the fixation cross was shown directly after participants were required to make a response and therefore removing the first 500 ms reduced the possibility of increased *mu* power during fixation as a result of participants moving their left hand back to a resting position after they had made their responses. This resulted in 160 fixation epochs for each participant that were each 1000 ms long.

EEG data were baseline corrected and band-pass filtered (1–30 Hz). Blink artefacts were detected by scanning the data from the EOG electrodes for voltages greater than 100 or − 100 µV. Once the blink artefacts were detected, these were corrected using covariance analysis (Curry Neuroimaging Suite 7, Compumetics Ltd, Australia). Any epochs that still contained non-cerebral artefacts (> 75 µV) were identified and removed from the analysis. Two participants were removed from the analysis (one participant from the high AQ group and one participant from the ASD group) because 62.5% or less of the epochs were valid for one or more of the individual conditions (mentalizing videos, non-mentalizing videos, fixation crosses in the mentalizing block, fixation crosses in the non-mentalizing block). Excluding these participants, only 5.6% of epochs were invalid across all participants. Preliminary analyses were carried out on the EEG data in order to clarify that the number of epochs that were excluded did not significantly differ between participant groups or experimental conditions. The numbers of excluded epochs were not normally distributed even after a log transformation was applied so non-parametric analyses were conducted. A Kruskal–Wallis test was used to investigate group differences in the number of epochs excluded and a Friedman’s ANOVA was used to identify differences in the number of excluded epochs across experimental conditions.

A Fast Fourier Transform (FFT) was used to calculate *mu* power in both the low alpha frequency range (8–10 Hz) and high alpha frequency range (10–12 Hz) during all epochs. The majority of previous studies investigating MS activity using EEG have used activity in the entire alpha frequency band (8–12/13 Hz) as a measure of *mu* power (Andrews et al. 2015; Oberman et al. 2005, 2008; Perry et al. 2011; Ulloa and Pineda 2007). However, there is accumulating evidence to suggest that lower (8–10 Hz) and higher (10–12 Hz) alpha bands reflect different processes and should therefore be analysed separately (Dumas et al. 2014; Frenkel-Toledo et al. 2014; Neuper et al. 2009; Pfurtscheller et al. 2000). Additionally, a previous study found reduced *mu* suppression in the 10–12 Hz range over frontal regions in adults with ASD but not the 8–10 Hz range when observing hand movements (Dumas et al. 2014). Consequently, lower and higher mu frequency bands were analysed separately in this study.

Average *mu* power in both frequency bands was then calculated for each epoch type (four epoch types: mentalizing videos, non-mentalizing videos, mentalizing fixation and non-mentalizing fixation) for every participant. The degree of *mu* suppression during each experimental condition was calculated by comparing average *mu* power during the video epochs compared to the fixation epochs in the same condition: [(fixation-video)/fixation]*100. Larger values indicated greater degrees of *mu* suppression. The *mu* suppression data for both frequency bands violated the assumption of normality so the data were log transformed. Log transformations cannot be carried out on negative values so a constant of 1300 was added to each data point to ensure that all values were positive before the log transformation. After the log transformation was applied, the data still violated the assumption of normality and therefore non-parametric analyses were conducted. Kruskal–Wallis tests were used to investigate group differences in *mu* suppression and related-samples Wilcoxon signed rank tests were carried out to investigate task-related changes in *mu* suppression. Analyses were carried out separately for both frequency bands.

### Eye-Tracking Analysis

**Fig. 2:**
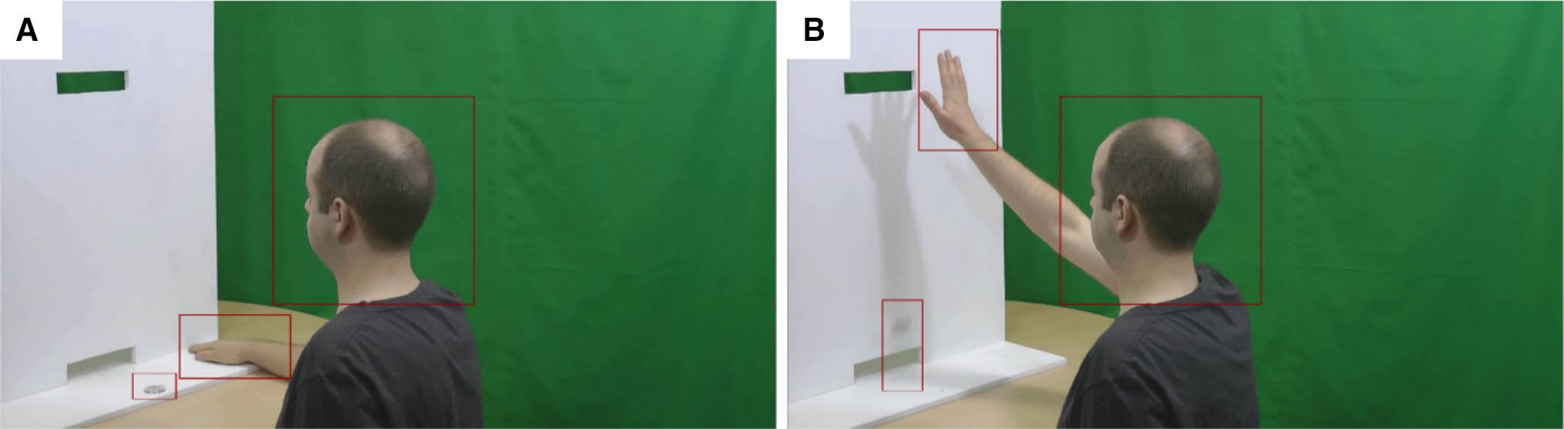
The dynamic regions of interest (ROIs) used in the eye-tracking data analysis for one of the action videos are shown overlaid onto screenshots from **a** the start and **b** the end of that particular video. Three dynamic ROIs corresponding to (1) The poker chip, (2) The actor’s head and (3) The actor’s hand, were created for each of the 120 videos individually

The eye-tracking data were analysed using EyeLink DataViewer software (SR Research Ltd., Ontario, Canada). Three dynamic rectangular regions of interest (ROIs) were created for each action video individually. These ROIs corresponded to the head of the actor, the actor’s hand and the poker chip (see Fig. 2). These three interest areas were chosen based on eye-tracking data from a previous behavioural study using the same stimuli (Cole et al. 2017). The total number and total duration of fixations in each ROI during each task (mentalizing/non-mentalizing) were calculated for each participant. ROIs were analysed separately as these data are not independent (participants cannot fixate in more than one ROI at once). The eye-tracking data were not normally distributed even after a log transformation was applied and therefore non-parametric analyses were conducted. Independent-samples Kruskal–Wallis tests were used to investigate group differences and related-samples Wilcoxon signed rank tests examined differences between tasks for each ROI.

### Additional Analyses

For all analyses (behavioural, EMG, EEG and eye-tracking), any significant task-related differences that were identified were investigated further by analysing the data collected during the presentation of clumsy actions across the two tasks. Identical clumsy actions were shown during both the mentalizing and non-mentalizing tasks. Analysing the data in this way eliminates the possibility that apparent effects of the task are due to differences in observed action kinematics.

Due to the spectral nature of ASD, any significant group differences that were found were also examined across the continuum of autistic traits. A principal components analysis (PCA) was performed on all the psychological test scores in order to obtain a single score for each participant that reflected the level of autistic traits that they displayed. Linear regression analyses were then used to examine whether the levels of autistic traits significantly predicted the outcome variables e.g. levels of *mu* suppression. These additional analyses were conducted to further support the relationships between the outcome variables and ASD.

Bayes factors were also calculated for all non-significant results to provide evidence for, or against, our hypotheses, irrespective of sample size (Dienes 2014). Bayes factors of 1 indicate equal amounts of evidence to support both the null hypothesis and the alternative hypothesis, higher Bayes factors indicate more evidence for the alternative hypothesis and lower values suggest more evidence for the null hypothesis. Bayes factors lower than 1/3 are considered to reflect substantial evidence for the null hypothesis and Bayes factors higher than 3 indicate substantial evidence for the alternative hypothesis (Dienes 2014).

## Results

### Psychological Tests

A one-way MANOVA identified that scores on all psychological tests (ADOS-2, AQ, EQ, TASIT and SRS-2) were significantly different between groups (see Table 3). Post-hoc pairwise comparisons identified that all groups were significantly different from each other on the ADOS-2, AQ and SRS-2 measures (Bonferroni correction applied). The Bonferroni corrected multiple comparisons identified that the high AQ and ASD groups did not significantly differ in TASIT scores (p = 0.92, B = 0.51). EQ scores did not significantly differ between low and high AQ groups (p = 0.08, B = 4.62), however, the Bayes factor indicated there was evidence for a difference in EQ score between low and high AQ groups. All other group comparisons were significant (p < 0.001 except difference in AQ scores between high AQ and ASD groups; p < 0.01, and TASIT scores between low and high AQ groups; p = 0.02). In all cases, where significant group differences were found, the ASD group had scores that reflected the highest level of autistic traits and the low AQ group had scores reflected the lowest level of autistic traits.

Principal component analysis (PCA) was conducted using the psychological test scores in order to obtain a single value for each participant that represented the level of autistic traits that they displayed. The psychological test scores correlated with each other (all rs > 0.35) meaning that they were suitable for PCA. The Kasier–Meyer–Olkin measure of sampling accuracy was 0.84 (above 0.6), Barlett’s test of sphericity was significant χ^2^(10) = 146.07, p < 0.001 and the communalities were all above 0.7 which collectively supported the inclusion of all the psychological tests in the PCA. PCA with varimax rotation was used. The initial eigenvalues from the PCA analysis showed that one factor (with an eigenvalue of 3.57) explained 71.36% of the variance in psychological test scores. No other factors had eigenvalues higher than Kaiser’s criterion of 1 and therefore only one factor was extracted. This factor was labelled ‘autistic traits’.

### EEG Data

#### 8–10 Hz

##### Group Differences

**Fig. 3:**
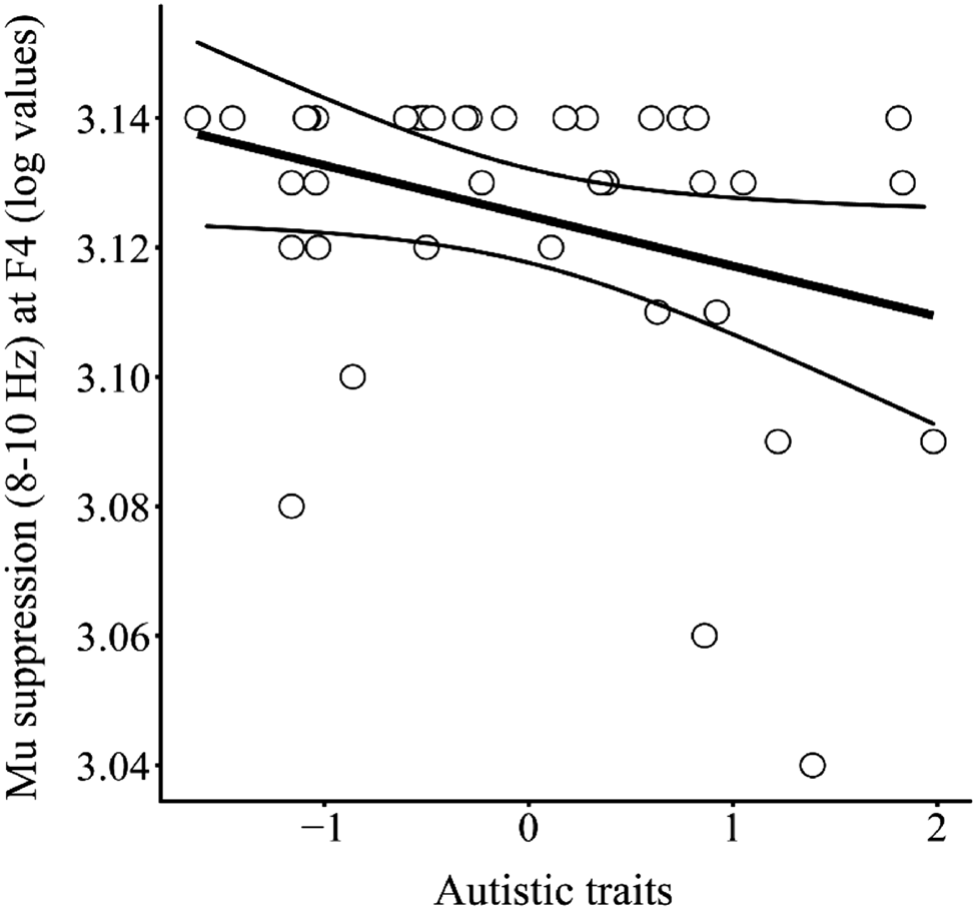
The relationship between the level of autistic traits that participants displayed and the level of *mu* suppression in the 8–10 Hz frequency range at F4. Levels of autistic traits significantly predicted the degree of *mu* suppression at F4; participants that exhibited higher levels of autistic traits showed lower levels of *mu* suppression (8–10 Hz) at F4 [F(1,38) = 0.47, p = 0.04, R^2^ = 0.11]. The curved lines represent 95% confidence intervals

There were significant differences in the levels of mu suppression in the 8–10 frequency band between groups during the mentalizing task at F4 (H(2) = 6.21, p < 0.05). Additionally, linear regression analysis demonstrated that the level of autistic traits that participants displayed significantly predicted the amount of *mu* suppression in 8–10 Hz band at F4 during the mentalizing task [F(1,38) = 0.47, p = 0.04, R^2^ = 0.11; see Fig. 3]. Pairwise-comparisons with adjusted p values (using the Bonferroni correction) are reported and demonstrate a borderline significant difference between the high AQ group and the ASD group (p = 0.05, r = 0.47; with lower levels of *mu* suppression in the ASD group). [After applying a Bonferroni correction, the new significance threshold was p = 0.017 (0.05/3)]. There were no significant differences between ASD and low AQ groups (p = 0.18, r = 0.37, B = 1.44) or the between the low and high AQ groups (p = 1.00, r = − 0.10, B = 0.46).

*Mu* suppression in the 8–10 Hz frequency band was significantly different between groups during the non-mentalizing task at F3 (H(2) = 10.10, p = 0.006) and FCZ (H(2) = 7.32, p = 0.03). Pairwise comparisons showed that the high AQ group displayed significantly lower levels of mu suppression than the low AQ group at F3 (p = 0.01, r = 0.54). No other group differences were significant once threshold significance values had been adjusted using the Bonferroni correction (p = 0.02; see supplementary material). Linear regression analysis demonstrated that the level of autistic traits that participants displayed was not a significant predictor of the amount of *mu* suppression in 8–10 Hz band during the non-mentalizing task at F3 [F(1,38) = 0.02, p = 0.90, R^2^ < 0.001, B = 0.44] or FCZ [F(1,38) = 0.03, p = 0.86, R^2^ < 0.01, B = 0.35].There were no other significant group differences in *mu* suppression in the 8–10 Hz frequency band (see Supplementary Material).

##### Task-Related Differences

Initial analyses identified that *mu* suppression in the 8–10 Hz band was significantly lower during the mentalizing task than the non-mentalizing task at F3 across all participants (T = 581, p = 0.02, r = 0.38). However, when this apparent significant task-related difference in *mu* suppression was investigated using data from the clumsy actions alone (in order to control for differences in action kinematics), there was no significant difference in *mu* suppression in the 8–10 Hz range at F3 between clumsy actions shown in the mentalizing task compared to the non-mentalizing task (T = 518, p = 0.15, r = 0.23, B = 0.23). There were also no task-related differences in *mu* suppression in the 8–10 Hz band at FCZ (T = 501, p = 0.21, r = 0.20, B = 0.22) or F4 (T = 495, p = 0.25, r = 0.18, B = 0.22).

#### 10–12 Hz

There were no significant differences in *mu* suppression in the 10–12 Hz frequency band between groups or across tasks at any of the cortical sites (see Supplementary Material).

### TMS Data

Across all participants, there was no significant difference in motor resonance values in the FDI muscle between the mentalizing and non-mentalizing tasks (F(1,32) = 0.30, p = 0.59, η_p_^2^ < 0.01), there was no significant interaction between participant group and the task (F(2,32) = 0.73, p = 0.49, η_p_^2^ = 0.04) and there were no significant group differences in motor resonance values (F(2, 32) = 0.73, p = 0.49, η_p_^2^ = 0.04). Bayesian t-tests indicated there was significant evidence against higher motor resonance values during the mentalizing task compared to the non-mentalizing task (B = 0.29). Bayes factors indicated that there was neither evidence for, nor against, group differences in motor resonance values; between low and high AQ groups (B = 0.83), between high AQ and ASD groups (B = 1.25) or between low AQ and ASD groups (B = 1.29).

There were no significant task or group differences for the ADM data (see Supplementary Material).

### Eye-Tracking

Across all participants, significantly more and longer fixations were made in the hand and head ROIs during the mentalizing task compared to the non-mentalizing task [hand ROI: number: (T = 169, p < 0.001, r = − 0.52), duration: (T = 288, p = 0.03, r = − 0.33); head ROI: number: (T = 271, p = 0.02, r = − 0.34), duration: (T = 344, p = 0.02, r = − 0.35)]. There was borderline significantly more fixations in the poker chip ROI during the mentalizing than the non-mentalizing task (T = 297, p = 0.05, r = 0.29). However, there was no significant task-related difference in the total duration of fixations within the poker chip ROI (T = 431, p = 0.61, r = − 0.08, B = 0.66).

When the eye-tracking data from the clumsy actions were analysed alone, all previously significant results (including the borderline significant difference) were still significant except for the duration of fixations within the head ROI (T = 344, p = 0.12, r = − 0.24, B = 0.97; see supplementary material for all results).There were no significant group differences in the eye-tracking data (see Supplementary Material).

### Relationships Between Data from Different Techniques

#### EEG and Behavioural Performance

**Fig. 4:**
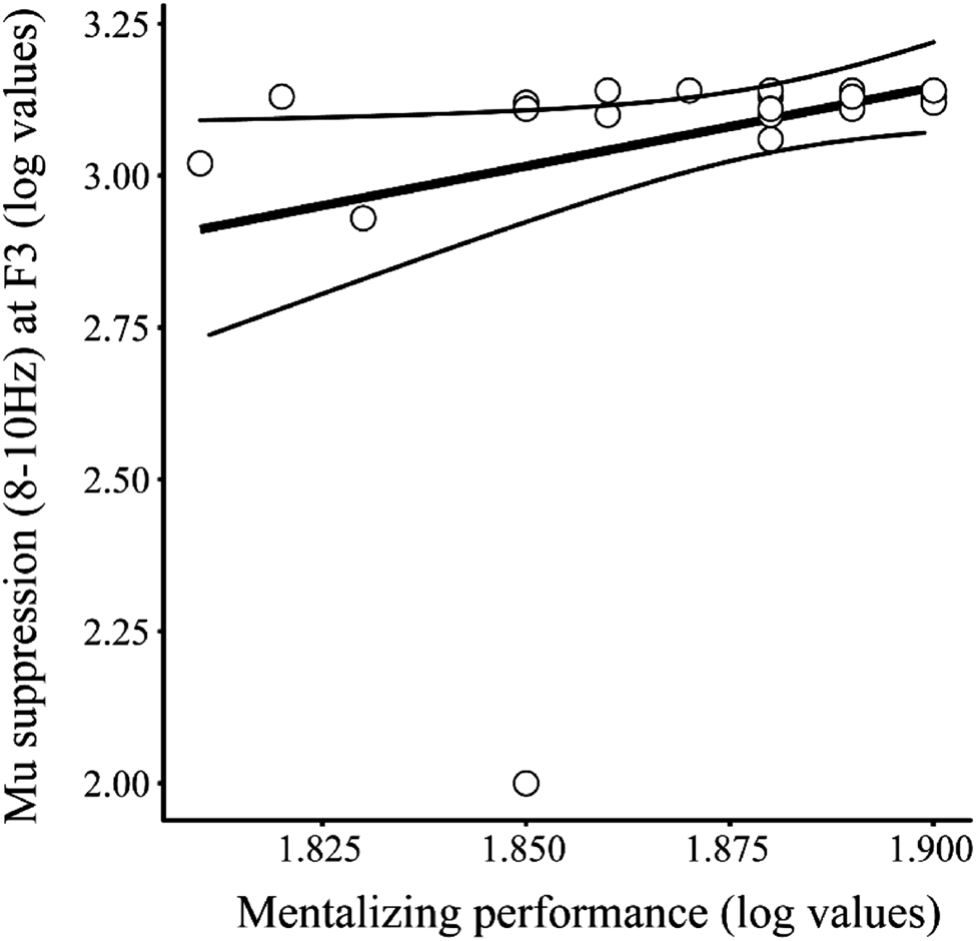
The relationship between performance on the mentalizing task and the level of *mu* suppression in the 8–10 Hz frequency band at F3. Mentalizing performance significantly predicted the degree of *mu* suppression at F3; participants with superior mentalizing performances also showed greater levels of *mu* suppression at F3 in the 8–10 Hz frequency band [F(1,38) = 5.64, p = 0.02, R^2^ = 0.13]. The curved lines represent 95% confidence intervals

When investigating the relationship between EEG and behavioural performances, linear regression analysis found that *mu* suppression in the 8–10 Hz frequency band at F3 during the mentalizing task significantly predicted performance on this task across all participants [F(1,38) = 5.64, p = 0.02, R^2^ = 0.13; see Fig. 4]. There were no other significant relationships between the EEG data and behavioural performance (see Supplementary Material).

#### Eye-Tracking and Behavioural Performance

The total duration of fixations within the poker chip ROI during the non-mentalizing task significantly predicted performance on the non-mentalizing task [F(1,41) = 5.14, p = 0.03, R^2^ = 0.11]. There were no other significant relationships between eye-tracking data and behavioural performance (see Supplementary Material).

#### Eye-Tracking and EEG

Linear regression analyses found that the degree of *mu* suppression did not significantly predict fixation patterns for any of the cortical sites (see Supplementary Material).

#### TMS and Other Measures

Linear regression analyses found that motor resonance values did not significantly predict behavioural performances or levels of *mu* suppression in either task (see Supplementary Material).

## Discussion

This study aimed to investigate the possible association between ASD and atypical MS activity when mentalizing, as well as the relationship between MS activity and mentalizing performance. Both TMS-induced MEPs and *mu* suppression (measured by EEG) were used as indices of MS activity. The EEG data show that higher levels of autistic traits (across clinical and non-clinical populations) were associated with lower levels of MS activation in the right hemisphere when mentalizing. These lower levels of MS activity in the right hemisphere were not associated with poorer mentalizing performances. In contrast, lower levels of MS activity in the left hemisphere were associated with poorer mentalizing performance but not associated with the levels of autistic traits that participants displayed. The TMS data did not show differences in MS activity between groups or a relationship between MS activity and mentalizing performances. Consequently, although our sample size is small, the EEG data provide evidence for MS involvement in mentalizing and reduced MS activity in adults with high levels of autistic traits. The different lateralisation of MS activity associated with task performance and MS activity associated with high levels of autistic traits means our data do not provide evidence that atypical MS functioning underlies mentalizing difficulties associated with ASD.

Across all participants, the level of autistic traits displayed significantly predicted levels of *mu* suppression in the 8–10 Hz frequency band at F4 during the mentalizing task (see Fig. 3). These data imply that high levels of autistic traits are associated with reduced MS activity in the right hemisphere when mentalizing. Our results support previous fMRI studies which have found reduced MS activation in adults with ASD during mentalizing tasks (Baron-Cohen et al. 1999; Hadjikhani et al. 2009; Holt et al. 2014; Kana et al. 2014; Wicker et al. 2008).

The lower levels of right mu suppression in individuals with high levels of autistic traits during the mentalizing task were not observed during the non-mentalizing task. These data suggest mentalizing induces atypical suppression of the right MS in these adults. Mentalizing tasks reliably induce activation in a cortical system known as the ‘mentalizing system’ (Ciaramidaro et al. 2014; de Lange et al. 2008; Lombardo et al. 2010; Spunt et al. 2011; Van Overwalle and Baetens 2009). Atypical connectivity between the mentalizing system and the MS has previously been reported in ASD (Damarla et al. 2010; Fishman et al. 2014; Just et al. 2007, 2004; Kennedy and Courchesne 2008; Noonan et al. 2009; Shih et al. 2010). It is possible that the reduced right MS activity we observed in adults with high levels of autistic traits was the result of atypical connectivity between the mentalizing system and the MS when inferring the intentions of others from their actions.

Although our EEG data suggest that MS activation in the right hemisphere is reduced in adults with high levels of autistic traits when mentalizing, no significant relationship was found between right-lateralised MS activity and mentalizing performance. Consequently, our data do not provide evidence that the atypical right MS activation identified underlies mentalizing difficulties associated with ASD. However, the lack of a significant relationship between MS activity and mentalizing performance may be due to compensatory strategies that individuals with high levels of autistic traits have adopted in order to successfully infer the intentions of others from action kinematics, despite atypical disengagement of the right MS. All participants in this study were high-functioning adults with IQ scores within the typical range (> 70). It is possible that if younger or lower functioning individuals were recruited they may not have developed sufficient compensatory mechanisms and a relationship between right mu suppression and mentalizing performance may have been found. Therefore, although our EEG data provide evidence against the broken mirror theory, the use of compensatory strategies as well as our small sample size may have contributed to the lack of a relationship between MS activation and mentalizing performance.

In contrast to the right-lateralised EEG data, left-lateralised mu suppression was not related to levels of autistic traits but was positively associated with mentalizing performance. Participants who exhibited superior performances on the mentalizing task also displayed higher levels of *mu* suppression in the 8–10 Hz frequency band at F3 during this task. These data support the motor resonance (or motor simulation) theory (Decety and Grèzes 2006; Landmann et al. 2011; Uithol et al. 2011). This theory states that observed actions are internally simulated in the observer’s own MS in order to infer the internal states of the individuals performing the actions. In our study, right-handed actions were viewed and therefore internal simulation of these actions would be predicted to result in particularly increased activation in left hemisphere motor areas (Aziz-Zadeh et al. 2002). The relationship found between MS activity and mentalizing performance in our EEG data supports the notion that internal simulation of observed hand actions by the contralateral MS is an important process in order to successfully infer others intentions. This compliments previous fMRI and TMS studies that have shown higher left MS activation when viewing social right-handed actions compared to those without social context (Becchio et al. 2012; Bucchioni et al. 2013; Enticott et al. 2013b) and the poorer mentalizing performances observed in patients with MS lesions (Besharati et al. 2016; Dal Monte et al. 2014).

Initial analyses of task-related differences in *mu* suppression suggested that left-lateralised MS activity was lower during the mentalizing task than the non-mentalizing task. However, this task-related difference in MS activity was eliminated when only identical (‘clumsy’) actions were analysed across tasks. This implies that the apparent task-related difference in MS activity in the left hemisphere was likely to be the product of differences in action kinematics between the videos shown across the mentalizing and non-mentalizing tasks. Determining the success of the successful actions shown in the non-mentalizing task required processing the actors’ hands returning to their side of the board without the poker chip. It is therefore likely that the successful actions were internally simulated for slightly longer periods of time than the spiteful actions shown in the mentalizing task, resulting in overall greater levels of MS activation during the non-mentalizing task.

Our EEG data show differences in *mu* suppression in the 8–10 Hz frequency band rather than the 10–12 Hz frequency band were associated with autistic traits and mentalizing performance. No significant relationships were found between *mu* suppression in the 10–12 Hz frequency band and any other measures. These data support previous EEG studies which have found *mu* suppression in the lower alpha frequency band (8–10 Hz) but not the higher alpha frequency band during action observation (Cochin 1999; Simon and Mukamel 2016). These EEG data also support the functional segregation of *mu* rhythm into two discrete sub-bands, complimenting previous work that found distinct *mu* responses in low and high alpha bands (Dumas et al. 2014; Frenkel-Toledo et al. 2014; Neuper et al. 2009; Pfurtscheller et al. 2000). The majority of previous studies investigating mu rhythm in individuals with ASD have not split mu rhythm into sub-bands (Raphael Bernier et al. 2013; Dumas et al. 2014; Fan et al. 2010; Oberman et al. 2005, 2008; Raymaekers et al. 2009). A previous EEG study that did investigate *mu* suppression in two discrete sub-bands in adults with ASD found reduced *mu* suppression in the 11–13 Hz frequency band when passively observing hand actions and no atypicalities in the 8–10 Hz frequency band (Dumas et al. 2014). Similar to this previous study, our data show no atypicalities in *mu* suppression in the 8–10 Hz range in adults with ASD when performing a non-mentalizing task. The reduced *mu* suppression in the upper sub-band during passive action observation in the previous study may be the result of the slightly higher frequency band used. This frequency band encroaches into the beta frequency range 12(/13)-30(/35)Hz (Haenschel et al. 2000; Kilavik et al. 2013; Miller 2007). Similar to *mu* suppression, oscillatory activity in the beta frequency range is suppressed when observing biological motion (Babiloni et al. 2002; Milston et al. 2013; Perry et al. 2010) and atypical oscillatory activity in the beta frequency range has previously been reported in adults with ASD (Cooper et al. 2013; Honaga et al. 2010). In summary, mu suppression in the 8–10 Hz frequency sub-band (and not 10–12 Hz) appears to reflect MS activity in the left hemisphere that is related to mentalizing performance, and MS activity in the right hemisphere is reduced in adults with high levels of autistic traits when mentalizing.

The TMS data show no relationship between motor resonance values and either mentalizing performance or autistic traits and no differences in motor resonance values across tasks. We had expected larger motor resonance values during the mentalizing task (Enticott et al. 2013b), and reduced task-related differences in motor resonance values in adults with high levels of autistic traits (Enticott et al. 2012; Puzzo et al. 2009; Théoret et al. 2005). TMS stimulation was applied to the left hemisphere meaning that motor resonance values reflected left MS activity. The lack of a relationship between autistic traits and motor resonance values as well as no task-related difference in motor resonance values complement our left-lateralised EEG data, when differences in action kinematics were controlled for. However, our TMS data did not replicate the relationship found between left MS activation and mentalizing performance in the EEG data.

A possible reason for the inconsistency between the TMS data and the EEG data is that these methods measure different aspects of MS functioning. Across all participants, motor resonance values did not significantly predict the degree of *mu* suppression (see Supplementary Material) supporting previous studies (Andrews et al. 2015; Lepage et al. 2008). Results from both MEG studies (Cheyne et al. 2003; Jones et al. 2009) and a combined MRI-EEG study (Arnstein et al. 2011) suggest that *mu* rhythms correspond to activation in S1. Although S1 is not considered to be a ‘core region’ of the MS, S1 has been reliably shown to display mirror properties (Confalonieri et al. 2012; Gazzola and Keysers 2009; Molenberghs et al. 2012; Porro et al. 1996). TMS on the other hand, is very unlikely to cause MEPs in muscles of the hand through means other than the stimulation of M1 (Lepage et al. 2008). TMS-induced MEPs measured during action observation are thought to measure increased excitability in M1 as the result of excitatory cortico-cortical connections from prefrontal MS areas (IFG/vPMC; Fadiga et al. 2005; Loporto et al. 2011). Therefore, if *mu* suppression measured by EEG reflects MS activity in S1 and TMS-induced MEPs provide an index of prefrontal MS activity then this could explain the differences between the results from these two measures.

An alternative reason for the inconsistency between the EEG and TMS data could be due to the differences in the spatial and temporal properties of these two measurements of MS activity. The EEG measurements in this study reflect the sum of post-synaptic neuronal activity over a large cortical area and a relatively long time period (throughout video or fixation cross display) whereas TMS measures brief induced increases in corticospinal activity from peripheral muscles, induced by stimulating a relatively small population of neurons at a discrete time point (Andrews et al. 2015; Pineda 2005; Rossini et al. 1994). Therefore, the EEG and TMS datamay differ due to differences in the spatial and temporal properties of the measurements.

The total duration of fixations in the poker chip ROI predicted non-mentalizing task performance but there were no significant relationships between any of the eye-tracking measures and mentalizing performance. This implies that the visual information within the poker chip ROI was vital for performance on the non-mentalizing task; this is to be expected as performances relied upon identifying whether the poker chip was successfully passed to another player or was dropped before being passed to another player. In contrast, during the mentalizing task, the final location of the poker chip was always the same (all actions were unsuccessful) but participants were required to infer the intentions of the actors from their action kinematics. The lack of any significant relationships between the eye-tracking data and mentalizing performance suggests that participants did not have a rigid method in which they did this. This is supported by the greater number of fixations made during the mentalizing task compared to the non-mentalizing task, suggesting a greater degree of re-diverting attention, perhaps reflecting an increased level of uncertainty regarding where to direct their visual attention.

There were no significant differences in the eye-tracking data associated with high levels of autistic traits during the mentalizing task. This means that the lower levels of MS activity during the mentalizing task exhibited by adults with high levels of autistic traits were not due to reduced fixation on the observed action kinematics in these individuals.

There are a number of limitations associated with this study including the small sample size, particularly for the TMS data, which may have resulted in limited power to detect differences in MS functioning associated with ASD. The particularly small sample size for the TMS data was due to a number of participants (n = 6) not being able to complete the TMS element of this study either due to not tolerating stimulation or having particularly high motor thresholds. This particularly small sample size may have contributed to the lack of differences in motor resonance values found both across tasks and between groups. The lack of significant mentalizing deficits in adults with high levels of autistic traits in our study may have also limited our ability to detect a relationship between motor resonance values and autistic traits; participants with higher levels of autistic traits and significant mentalizing deficits were recruited then a significant relationship may have been observed. However, previous studies have also reported typical motor resonance values in adults with ASD (Enticott et al. 2013b; Kirkovski et al. 2016) and the lower levels of mu suppression found in our study suggest MS atypicalities were detectable in our participant sample regardless of typical behavioural performances.

It is possible that mu suppression variability was higher both between participants and within individuals with high levels of autistic traits compared to those with low levels of autistic traits. Therefore, the reduced levels of right mu suppression observed in individuals with high levels of autistic traits when mentalizing may reflect intermittent displays of typical mu suppression rather than consistently reduced mu suppression. The investigation of individual and within group variability in mu suppression is beyond the scope of this study but could be an interesting avenue for future research. Although a significant relationship was found between autistic traits and right-lateralised mu suppression (8–10 Hz), significant group differences were not observed. Examining neural differences in terms of the continuous measure of autistic traits may have been a more sensitive measure than examining group differences due to within group variability in autistic traits, and therefore neural measures, may have reduced chances of observing group differences. The inclusion of medicated participants in this study may have also influenced the data; six participants in the ASD group were taking psychotropic medications which have been shown to increase corticospinal excitability (Gilbert et al. 2006; Minelli et al. 2010). Due to the high comorbidity of ADHD, depression and anxiety in ASD, the inclusion of adults taking psychotropic medication is common in TMS studies with ASD participants (Enticott et al. 2010, 2013a; Oberman et al. 2014). Despite the possible influence of psychotropic medication on corticospinal excitability, our TMS data show no group differences in resting motor thresholds and there was no significant difference in resting motor threshold values between medicated and non-medicated participants (see Supplementary Material). Despite these limitations, our EEG data add to the existing literature by identifying lower levels of right MS activity in adults with high levels of autistic traits when inferring the intentions of others from their actions and higher levels of left MS activity associated with superior mentalizing performances. These EEG data suggest that the MS has a role in inferring the intentions of others from their actions, providing support for the motor resonance theory of social cognition (Agnew et al. 2007; Landmann et al. 2011; Leslie et al. 2004; Rizzolatti et al. 2002). Additionally, adults with high levels of autistic traits appear to display atypical top-down suppression from the mentalizing system to the MS in the right hemisphere when inferring the intentions of others. Therefore, this study provides evidence for reduced MS activity in adults with high levels of autistic traits when mentalizing and a potential role of the MS in inferring the intentions of others.

## Electronic Supplementary material

Below is the link to the electronic supplementary material.

Supplementary material 1 (DOCX 30 KB)
