# Supplementary material for: Investigating Mirror System (MS) Activity in Adults with ASD When Inferring Others’ Intentions Using Both TMS and EEG
Source: J Autism Dev Disord. 2018 Feb 16;48(7):2350–67. doi: 10.1007/s10803-018-3492-2 (PMC5996018; doi:10.1007/s10803-018-3492-2)
Supplement: Supplementary file 1 — Supplementary material 1 (DOCX 30 KB) [file 10803_2018_3492_MOESM1_ESM.docx]

# Supplementary material

## Task instructions

You will watch short video clips of people playing a poker chip game; each video will be 4 seconds long. Each video clip will show an actor either successfully or unsuccessfully passing a poker chip through slots in a board to someone on the other side, who is out of view. After each video you will be shown a response screen asking you to decide whether the poker chip was successfully passed through the board or not (successful or unsuccessful) or whether the poker chip was accidentally or deliberately dropped (clumsy or spiteful). In the poker chip game, if the actor deliberately doesn’t pass the poker chip, this is a ‘spiteful’ action because the other player wants the poker chip. If the actor accidentally drops the poker chip, this would be a ‘clumsy’ action. You will indicate your decision by pressing ‘1’ or ‘2’ on the computer keyboard. ‘1’ always corresponds to the first option shown on the screen and ‘2’ always corresponds with the second option shown on the screen. So it would be clumsy (shows pressing number ‘1’) or spiteful (demonstrates pressing number ‘2’) or successful (shows pressing number ‘1’) or unsuccessful (demonstrates pressing number ‘2’). I will let you know at the start of each task which decision you will be asked to make after each video; it will be the same decision after every video until we switch tasks. We will have a short break after the first task before we start the next one. Each task will last about 10 minutes. I will give you some practice trials now of each task so you will know what both tasks will be like, let me know if you have any questions.

## Behavioural data

There were no significant group differences in behavioural performance in either the mentalizing (H(2)=1.92, p=.38) or the non-mentalizing task (H(2)=4.70, p=.10). Bayesian t-tests provided evidence neither evidence for, nor against differences in behavioural performance between Low AQ and high AQ groups during the mentalizing task (B=.54) but substantial evidence against differences in performance between high AQ and ASD (B=.02), low AQ and ASD (B=.15) on the mentalizing task. There was also substantial evidence against group differences on the non-mentalizing task [Low AQ and high AQ: B=.12; high AQ and ASD: B=.07; low AQ and ASD: B= .15]. Across all participants, performance was significantly poorer on the mentalizing task than the non-mentalizing task (T=537, p<.001, r=.70).

## TMS data

### Preliminary analysis of the TMS data

There were no group differences in motor threshold values, no significant differences in baseline levels of corticospinal excitability before and after the experiment and no differences in the number of excluded MEPs both between groups and tasks (see below).

### Motor thresholds

A one-way ANOVA showed that the 1mv motor thresholds were not significantly different between low AQ (M=49.47, SD=4.9), high AQ (M=51.29, SD=10.36) and ASD (M=45.89, SD=7.40) groups [F(2, 35)=1.29, p=.29, η_p_^2^=.07].

An independent samples t-test identified that within the ASD group, motor threshold values were not significantly different between medicated (M=44.00, SD=4.90) and non-medicated (M=47.50, SD=10.66) participants [t(6)=.60, p=.57].

### Differences in Baseline Corticospinal Excitability

Three participants (one from each group) were not included in this section of the analysis because their post-experiment baseline corticospinal excitability measures were invalid (>50% MEPs in the FDI muscle <.2mV). For two of the participants this was due to the ‘hot spot’ for the FDI being particularly difficult to maintain and the post-experiment baseline was missing for one participant due to an error when acquiring the data.

A (3 x 2) repeated measures ANOVA found that baseline MEP sizes in the FDI muscle were not significantly different before and after the experiment across all participants (F(1, 30)=3.62, p=.07, η_p_^2^=.11), there were no significant differences in baseline MEP sizes between groups (F(2, 30)=.56, p=.58, η_p_^2^=.04) and there was no significant interaction between participant group and the time point in which baseline measures were taken (F(2, 30)=.18, p=.84, η_p_^2^=.01).

There were no significant group differences in pre-experiment (H(2)=2.23, p=.33) and post-experiment (H(2)=.53, p=.77) baseline MEP sizes in the ADM muscle. Additionally, baseline MEPs in the ADM muscle were not significantly different before and after the experiment (T=218, p=.26, r=-.19) across all participants.

### Excluded MEPs

There were no significant group differences in the number of excluded MEPs in the mentalizing task[H(2)=1.60, p=.45] or the non-mentalizing task [H(2)=3.59, p=.17].

There was also no significant difference in the number of MEPs that were excluded between the two tasks across all participants [T=78, p=.49, r=-.12].

### ADM data

Motor resonance in the ADM muscle was not significantly different between the mentalizing and non-mentalizing tasks [T=374, p=.33, r=.16, B=.34]. There were also no group differences in ADM motor resonance values in the mentalizing task [H(2)=.96, p=.62] or the non-mentalizing task [H(2)=1.67, p=.43]. Bayesian t-tests indicated there was neither evidence for, nor against, group differences in ADM motor resonance values during the mentalizing task [between low and high AQ groups (B=1.28), between high AQ and ASD groups (B=.96) or between low AQ and ASD groups (B=1.18)] or the non-mentalizing task [between low and high AQ groups (B=1.35), between high AQ and ASD groups (B=.87) or between low AQ and ASD groups (B=1.02)]

## EEG data

### Excluded Epochs

There was a significant group difference in the number of video epochs excluded from the non-mentalizing task [H(2)=8.79, p=.01]. Pairwise comparisons with adjusted p values identified significantly more non-mentalizing video epochs were excluded in the low AQ group than the ASD group (p=.019) but there were no other significant group differences (High AQ & ASD groups: p=1.00; low & high AQ groups: p=.06).

There were no significant group differences in the number of epochs excluded for any of the other epoch types [mentalizing videos: H(2)=3.35, p=.19, fixation cross in the mentalizing task: H(2)=1.93, p=.38, fixation cross in the non-mentalizing task: H(2)=2.66, p=.26].

Across all participants, there were significant differences in the number of epochs excluded between epoch types [χ^2^(3)=16.01, p<.01]. Subsequent Wilcoxon tests found that a larger number of non-mentalizing video epochs were excluded compared to the mentalizing video epochs (T=51.01, p<.01, r=.50), the fixation cross epochs in the mentalizing task (T=54.50, p<.001, r=-.58) or fixation epochs in the non-mentalizing task (T=119, p<.01, r=-.48). There were no other significant differences in the number of epochs excluded between different epoch types (mentalizing videos vs mentalizing fixation: T=198.50, p=.14, r=-.23; mentalizing fixation vs non-mentalizing fixation: T=309.50, p=.39, r=.13; mentalizing videos vs non-mentalizing fixation: T=260, p=.52, r=-.10).

### 8-10Hz

Pairwise comparisons with adjusted p-values showed that *mu* suppression in the 8-10Hz range at F3 during the non-mentalizing task was significantly lower in the high AQ group than the ASD group (p=.03, r=-.51) or the low AQ group (p=.01, r=.54). There was no significant group difference between the low AQ group and the ASD group (p=1.00, r=.02, B=1.16).

*Mu* suppression in the 8-10Hz range during the non-mentalizing task at FCz was significantly lower in the high AQ group than the ASD group (p=.04, r=-.49; Bonferroni correction applied). Levels of mu suppression were not significantly different between the low & high AQ groups (p=.10, r=.40, B=1.61) or the low AQ group and the ASD group (p=1.00, r=-.09, B=.42) at FCz during the non-mentalizing task.

There were no significant group differences in mu suppression during the mentalizing task at F3: H(2)=.34, p=.84, FCZ: H(2)=5.65, p=.06 or during the non-mentalizing task at F4: H(2)=3.38, p=.19]. Bayesian t-tests indicated that there was neither evidence for, nor against, group differences at F3 [low and high AQ: B=1.36; high AQ & ASD: B=.41, Low AQ and ASD: B=1.28] or FCZ [low and high AQ: B=1.26; high AQ & ASD: B=1.17, Low AQ and ASD: B=1.43] during the mentalizing task or at F4 during the non-mentalizing task [low and high AQ: B=1.54; high AQ & ASD: B=.36, Low AQ and ASD: B=.42].

### 10-12Hz

There were no significant group differences in *mu* suppression in the 10-12Hz frequency band during either task at any of the cortical sites [mentalizing task: F3: H(2)=.92, p=.63; FCZ: H(2)=.10, p=.95; F4: H(2)=.94, p=.62; non-mentalizing task: F3: H(2)=.42, p=.81; FCZ: H(2)=.58, p=.75; F4: H(2)=.82, p=.67]. Bayesian t-tests showed that there was neither evidence for, nor against, group differences at F3 [low and high AQ: B=.39; high AQ & ASD: B=1.29, Low AQ and ASD: B=1.14], FCZ [low and high AQ: B=.40; high AQ & ASD: B=1.39, Low AQ and ASD: B=1.17] or F4 [low and high AQ: B=.40; high AQ & ASD: B=1.44, Low AQ and ASD: B=1.18] during the mentalizing task. Similarly, Bayesian t-tests indicated neither evidence for, nor against, group differences during the non-mentalizing task: F3 [low and high AQ: B=.40; high AQ & ASD: B=.94, Low AQ and ASD: B=.42], FCZ [low and high AQ: B=.46; high AQ & ASD: B=.41, Low AQ and ASD: B=.41], F4 [low and high AQ: B=1.19; high AQ & ASD: B=.40, Low AQ and ASD: B=.40]. There were also no significant differences in *mu* suppression in the 10-12Hz band between tasks at any of the cortical sites across all participants (F3: T=526, p=.12, r=.24, B=.19; FCZ: T=511, p=.18, r=.21, B=19.; F4: T=404, p=.94, r=-.13, B=.20).

## Eye-tracking data

### Hand ROI

When task-related differences were investigated using data from the clumsy actions alone, the number (T=229, p<.01, -.45) and duration (T=264, p=.01, -.38) of fixations made during the mentalizing task were still significantly higher than the non-mentalizing task.

There were no significant group differences in the number or duration of fixations within the hand ROI in the mentalizing task[number of fixations: H(2)=1.89, p=.39; duration: H(2)=3.59, p=.17] or the non-mentalizing task[number of fixations: H(2)=2.25, p=.32; duration: H(2)=4.21, p=.12]. Bayesian t-tests indicated that there was neitherevidence for, nor against group differences in the number and duration of fixations within the hand ROI during the mentalizing task [No. of fixations: low AQ vs high AQ: B=.64; high AQ vs ASD:B=.78; low AQ vs ASD; B=.78; total duration: low AQ vs high AQ: B=.74; high AQ vs ASD: B=.82; low AQ vs ASD: B=.87]

Non-mentalizing task [No. of fixations: low AQ vs high AQ: B=.63; high AQ vs ASD:B=.79.; low AQ vs ASD; B=.76.; total duration: low AQ vs high AQ: B=.63; high AQ vs ASD: B=.85; low AQ vs ASD: B=.82]

### Head ROI

When task-related differences were investigated using data from the clumsy actions alone, significantly more fixations were made within the head ROI during the mentalizing task (T=284, p=.02, r=-.35) but the total duration of fixations in the head ROI was not significantly different between tasks (T=344, p=.12, r=-.24, B=1.56).

There were no significant group differences in the number or duration of fixations within the head ROI in the mentalizing task [number of fixations: H(2)=.82, p=.66; duration: H(2)=1.46, p=.48] or the non-mentalizing task [number of fixations: H(2)=3.10, p=.21; duration: H(2)=3.49, p=.17]. Bayesian t-tests indicated that there was no evidence for, nor against group differences in the number and duration of fixations within the head ROI during the mentalizing task [No. of fixations: low AQ vs high AQ: B=.76; high AQ vs ASD:B=.78; low AQ vs ASD; B=.65; total duration: low AQ vs high AQ: B=.80; high AQ vs ASD: B=.79; low AQ vs ASD: B=.68] and the non-mentalizing task [No. of fixations: low AQ vs high AQ: B=.89; high AQ vs ASD:B=.71; low AQ vs ASD; B=.87; total duration: low AQ vs high AQ: B=.87; high AQ vs ASD: B=.71; low AQ vs ASD: B=.86].

### Poker Chip ROI

When the borderline significant difference in the number of fixations in the poker chip ROI was investigated using the clumsy actions alone this difference was significant (T=278, p=.03, r=-.33; significantly more fixations were made during the mentalizing task than the non-mentalizing task).

There were no significant group differences in the number or duration of fixations within the poker chip ROI in the mentalizing task [number of fixations: H(2)=1.45, p=.48; duration: H(2)=.65, p=.72] or the non-mentalizing task[number of fixations: H(2)=1.79, p=.41; duration: H(2)=1.81, p=.41]. Bayesian t-tests indicated that there was no evidence for, nor against group differences in the number and duration of fixations within the head ROI during the mentalizing task [No. of fixations: low AQ vs high AQ: B=.81; high AQ vs ASD:B=.93; low AQ vs ASD; B=.82; total duration: low AQ vs high AQ: B=74.; high AQ vs ASD: B=.87; low AQ vs ASD: B=.82] and the non-mentalizing task [No. of fixations: low AQ vs high AQ: B=.67; high AQ vs ASD:B=.73; low AQ vs ASD; B=.73; total duration: low AQ vs high AQ: B=.76; high AQ vs ASD: B=.70; low AQ vs ASD: B=.77].

## Relationships between data from different techniques

### TMS and behavioural performance

Motor resonance values for the FDI muscle during the mentalizing task[F(1,33)=.11, p=.75, R^2^<.01, B=.17] and the non-mentalizing task[F(1,33)=.91, p=.35, R^2^=.03, B=.24] did not significantly predict performances on these tasks.

### EEG and behavioural performance

The degree of *mu* suppression in the 8-10Hz frequency band at the other cortical sites and the 10-12Hz frequency band at all cortical sites during the mentalizing task did not significantly predict mentalizing performance [8-10Hz at FCZ: F(1,38)=2.77, p=.10, R^2^=.07, B=1.69; F4: F(1,38)=3.61, p=.07, R^2^=.09, B=2.46; 10-12Hz: F3: F(1,38)=.23, p=.63, R^2^<.01, B=.39; FCZ: F(1,38)=.40, p=.53, R^2^=.01, B=.46; F4: F(1,38)=.44, p=.51, R^2^=.01, B=.47].

Performances on the non-mentalizing task were not significantly predicted by the degree of *mu* suppression at any cortical site in either frequency band [8-10Hz: F3: F(1,38)=3.26, p=.08, R^2^=.08, B=2.11; FCZ: F(1,38)=1.98, p=.17, R^2^=.05, B=1.16; F4: F(1,38)=.76, p=.39, R^2^=.02, B=.59; 10-12Hz: F3: F(1,38)=.03, p=.87, R^2^<.01, B=.29; FCZ: F(1,38)=.05, p=.83, R^2^<.01, B=.31; F4: F(1,38)=.16, p=.69, R^2^<.01, B=.36].

### EEG and TMS

FDI motor resonances during the mentalizing task did not predict levels of *mu* suppression at any of the cortical sites in either frequency band: 8-10Hz: F3:[F(1,30)=.17, p=.68, R^2^<.01, B=.33] FCZ: [F(1,30)=.48, p=.50, R^2^=.02, B=.28] F4: [F(1,30)<.01, p=.96, R^2^<.01, B=.45]; 10-12Hz: F3:[F(1,30)=.22, p=.65, R^2^<.01, B=.62] FCZ: [F(1,30)=.17, p=.69, R^2^<.01, B=.59] F4: [F(1,30)=.21, p=.65, R^2^<.01, B=.62].

FDI motor resonances during the non-mentalizing task also did not predict levels of *mu* suppression at any of the cortical sites in either frequency band: 8-10Hz: F3: [F(1,30)=.25, p=.62, R^2^<.001, B=.64] FCZ: [F(1,30)=.42, p=.52, R^2^=.01, B=.29] F4: [F(1,30)<.001, p=.99, R^2^<.001, B=.44]; 10-12Hz: F3:[F(1,30)=.05, p=.82, R^2^<.01, B=.51 FCZ: [F(1,30)=.02, p=.90, R^2^<.01, B=.48] F4: [F(1,30)=.06, p=.80, R^2^<.01, B=.52].

### Eye-tracking and behavioural

Mentalizing performances were not predicted by the number of fixations or total duration of fixations within any of the ROIs during the mentalizing task [number of fixations: hand [F(1,41)=1.67, p=.20, R^2^=.04, B=.91]; head [F(1,41)=1.43, p=.24, R^2^=.03, B=.96]; poker chip [F(1,41)=.06, p=.82, R^2^<.01, B=.61]; duration of fixations: hand [F(1,41)=1.90, p=.18, R^2^=.04, B=.99]; head [F(1,41)=1.04, p=.31, R^2^=.03, B=86]; poker chip [F(1,41)=.14, p=.72, R^2^<.01, B=.62].

The total duration of fixations within the ROIs other than the poker chip ROI and the number of fixations within all the ROIs during the non-mentalizing task did not significantly predict performance on the non-mentalizing task [duration: hand F(1,41)=.54, p=.47, R^2^=.01, B=.74; head F(1,41)=.20, p=.65, R^2^<.01, B=.92; number of fixations: hand F(1,41)=.81, p=.38, R^2^=.02, B=.80; head [F(1,41)=.01, p=.91, R^2^<.001, .97 and poker chip F(1,41)=2.08, p=.16, R^2^=.05, B=1.01].

### Eye-tracking & EEG

### 8-10Hz

*Mu* suppression (8-10Hz) at F3 during the mentalizing task did not significantly predict the number or duration of fixations in any of the ROIs [number of fixations: hand: [F(1,38)<.01, p=.97, R^2^<.001, B=.21]; head: [F(1,38)<.01, p=.97, R^2^<.001, B=.21]; poker chip: [F(1,38)=.16, p=.67, R^2^<.01, B=.15]; total duration of fixations: hand: [F(1,38)<.01, p=.96, R^2^<.001, B=.19]; head: [F(1,38)<.01, p=.94, R^2^<.001, B=.19]; poker chip: [F(1,38)=.97, p=.33, R^2^=.03, B=.11].

*Mu* suppression (8-10Hz) at F3 during the non-mentalizing task did not significantly predict the number or duration of fixations in any of the ROIs [number of fixations: hand: [F(1,38)=.28, p=.60, R^2^<.01, B=.33]; head: [F(1,38)=.25, p=.62, R^2^<.01, B=.32]; poker chip: [F(1,38)<.01, p=.98, R^2^<.001, B=.20]; total duration of fixations: hand: [F(1,38)=.16, p=.69, R^2^<.01, B=.29]; head: [F(1,38)=.50, p=.49, R^2^=.01, B=.39]; poker chip: [F(1,38)=.08, p=.78, R^2^<.01, B=.25].

*Mu* suppression (8-10Hz) at F4 during the mentalizing task did not significantly predict the number or duration of fixations in any of the ROIs [number of fixations: hand: [F(1,38)=.04, p=.84, R^2^<.01, B=.17]; head: [F(1,38)=1.63, p=.21, R^2^=.04, B=.80]; poker chip: [F(1,38)=.43, p=.52, R^2^=.01, B=.02]; total duration: hand: [F(1,38)<.001, p=.97, R^2^<.001, B=.20]; head: [F(1,38)=.63, p=.43, R^2^=.02, B=.44]; poker chip: [F(1,38)<.01, p=.95, R^2^<.001, B=.19].

*Mu* suppression (8-10Hz) at F4 during the non-mentalizing task did not significantly predict the number or duration of fixations in any of the ROIs [number of fixations: hand: [F(1,38)=.08, p=.79, R^2^<.01, B=.16]; head: [F(1,38)=.31, p=.58, R^2^<.01, B=.33]; poker chip: [F(1,38)=.07, p=.80, R^2^<.01, B=.25]; total duration of fixations: hand: [F(1,38)=.03, p=.86, R^2^<.01, B=.23]; head: [F(1,38)=.45, p=.51, R^2^=.01, B=.38]; poker chip: [F(1,38)=.08, p=.79, R^2^<.01, B=.25].

*Mu* suppression (8-10Hz) at FCZ during the mentalizing task did not significantly predict the number or duration of fixations in any of the ROIs [number of fixations: hand: [F(1,38)=.44, p=.51, R^2^=.01, B=.13]; head: [F(1,38)=1.06, p=.31, R^2^=.03, B=.14]; poker chip: [F(1,38)=2.92, p=.10, R^2^=.07, B=.08]; total duration of fixations: hand: [F(1,38)=.04, p=.85, R^2^<.01,B=.17]; head: [F(1,38)=.47, p=.50, R^2^=.01, B=.38]; poker chip: [F(1,38)=2.04, p=.16, R^2^=.05, B=.08].

*Mu* suppression (8-10Hz) at FCZ during the non-mentalizing task did not significantly predict the number or duration of fixations in any of the ROIs [number of fixations: hand: [F(1,38)=.28, p=.60, R^2^<.01, B=.14]; head: [F(1,38)=.04, p=.84, R^2^<.01, B=.17]; poker chip; [F(1,38)<.01, p=.95, R^2^<.01, B=.19]; total duration of fixations: hand: [F(1,38)=.14, p=.71, R^2^<.01, B=.15]; head: [F(1,38)=.18, p=.67, R^2^<.01, B=.29]; poker chip: [F(1,38)=.05, p=.83, R^2^<.01, B=.24].

### 10-12Hz

*Mu* suppression (10-12Hz) at F3 during the mentalizing task did not significantly predict the number or duration of fixations in any of the ROIs [number of fixations: hand: [F(1,38)=.04, p=.85, R^2^<.01, B=.23]; head: [F(1,38)=.13, p=.72, R^2^<.01, B=.28]; poker chip: [F(1,38)=.49, p=.49, R^2^=.01, B=.12]; total duration of fixations: hand: [F(1,38)=.11, p=.74, R^2^<.01, B=.26]; head: [F(1,38)<.01, p=.98, R^2^<.001, B=.20]; poker chip: [F(1,38)=.62, p=.44, R^2^=.02, B=.12].

*Mu* suppression (10-12Hz) at F3 during the non-mentalizing task did not significantly predict the number or duration of fixations in any of the ROIs [number of fixations: hand: [F(1,38)=.67, p=.42, R^2^=.02, B=.11]; head: [number of fixations: hand: [F(1,38)=1.82, p=.19, R^2^=.05, B=.09]; poker chip: [F(1,38)=.19, p=.67, R^2^<.01, B=.15]; total duration of fixations: hand: [F(1,38)=.73, p=.40, R^2^=.02, B=.47]; head: [F(1,38)=1.64, p=.21, R^2^=.04, B=.84]; poker chip: [F(1,38)=.06, p=.81, R^2^<.01, B=.17].

*Mu* suppression (10-12Hz) at FCZ during the mentalizing task did not significantly predict the number or duration of fixations in any of the ROIs [number of fixations: hand: [F(1,38)=.27, p=.60, R^2^<.01, B=.14]; head: [F(1,38)=.57, p=.45, R^2^=.02, B=.42]; poker chip: [F(1,38)=3.49, p=.07, R^2^=.08, B=.07]; total duration of fixations: hand: [F(1,38)=.16, p=.69, R^2^<.01, B=.15]; head: [F(1,38)=.02, p=.89, R^2^<.01, B=.22]; poker chip: [F(1,38)=3.94, p=.05, R^2^=.09, B=.07].

*Mu* suppression (10-12Hz) at FCZ during the non-mentalizing task did not significantly predict the number or duration of fixations in any of the ROIs [number of fixations: hand: [F(1,38)=.54, p=.47, R^2^=.01, B=.41]; head: [F(1,38)=.04, p=.84, R^2^<.01, B=.17]; poker chip: [F(1,38)=.03, p=.86, R^2^<.01, B=.17]; total duration of fixations: hand: [F(1,38)=.6`, p=.44, R^2^=.02, B=.12]; head: [F(1,38)=.10, p=.76, R^2^<.01, B=.16]; poker chip: [F(1,38)=.21, p=.65, R^2^<.01, B=.14].

*Mu* suppression (10-12Hz) at F4 during the mentalizing task did not significantly predict the number or duration of fixations in any of the ROIs [number of fixations: hand: [F(1,38)<.01, p=.93, R^2^<.001, B=.19]; head: [F(1,38)=.68, p=.41, R^2^=.02, B=.45]; poker chip: [F(1,38)=1.31, p=.26, R^2^=.03, B=.10]; total duration of fixations: hand: [F(1,38)<.01, p=.98, R^2^<.001, B=.20]; head: [F(1,38)=.09, p=.76, R^2^<.01, B=.26]; poker chip: [F(1,38)=1.42, p=.24, R^2^=.04, B=.10].

*Mu* suppression (10-12Hz) at F4 during the non-mentalizing task did not significantly predict the number or duration of fixations in any of the ROIs [number of fixations: hand: [F(1,38)=.62, p=.44, R^2^=.02, B=.12]; head: [F(1,38)=3.38, p=.07, R^2^=.08, B=2.1]; poker chip: [F(1,38)=.45, p=.51, R^2^=.01, B=.13]; total duration of fixations: hand: [F(1,38)=.85, p=.36, R^2^=.02, B=.11]; head: [F(1,38)=2.47, p=.12, R^2^=.06, B=1.31]; poker chip: [F(1,38)=.76, p=.39, R^2^=.02, B=.11].
